# Kinase Inhibitors in the Treatment of Ovarian Cancer: Current State and Future Promises

**DOI:** 10.3390/cancers14246257

**Published:** 2022-12-19

**Authors:** Aikaterini Skorda, Marie Lund Bay, Sampsa Hautaniemi, Alexandra Lahtinen, Tuula Kallunki

**Affiliations:** 1 Cancer Invasion and Resistance Group, Danish Cancer Society Research Center, Strandboulevarden 49, DK-2100 Copenhagen, Denmark; 2 Research Program in Systems Oncology, Research Programs Unit, Faculty of Medicine, University of Helsinki, FI-00014 Helsinki, Finland; 3 Department of Drug Design and Pharmacology, Faculty of Health and Medical Sciences, University of Copenhagen, DK-2200 Copenhagen, Denmark

**Keywords:** clinical trials, high-grade serous ovarian carcinoma, kinase inhibitor, patient-derived tumor organoids, patient-derived xenografts, personalized medicine

## Abstract

**Simple Summary:**

Ovarian cancer is the most lethal gynecological cancer. Currently there is no curative treatment for relapsed, standard treatment resistant ovarian cancer. Here we discuss and summarize recent clinical and preclinical studies concerning the possibility to use small molecule kinase inhibitors as a treatment of advanced platinum and taxane resistant ovarian cancer, with a focus on high grade serous ovarian cancer, the most common and most aggressive form of it. Some of these results seem rather promising and support for the feasibility of kinase inhibition as a personalized combinatory treatment. This will optimally require tumor sequencing, longitudinal sampling, and additional preclinical and clinical studies.

**Abstract:**

Ovarian cancer is the deadliest gynecological cancer, the high-grade serous ovarian carcinoma (HGSC) being its most common and most aggressive form. Despite the latest therapeutical advancements following the introduction of vascular endothelial growth factor receptor (VEGFR) targeting angiogenesis inhibitors and poly-ADP-ribose-polymerase (PARP) inhibitors to supplement the standard platinum- and taxane-based chemotherapy, the expected overall survival of HGSC patients has not improved significantly from the five-year rate of 42%. This calls for the development and testing of more efficient treatment options. Many oncogenic kinase-signaling pathways are dysregulated in HGSC. Since small-molecule kinase inhibitors have revolutionized the treatment of many solid cancers due to the generality of the increased activation of protein kinases in carcinomas, it is reasonable to evaluate their potential against HGSC. Here, we present the latest concluded and on-going clinical trials on kinase inhibitors in HGSC, as well as the recent work concerning ovarian cancer patient organoids and xenograft models. We discuss the potential of kinase inhibitors as personalized treatments, which would require comprehensive assessment of the biological mechanisms underlying tumor spread and chemoresistance in individual patients, and their connection to tumor genome and transcriptome to establish identifiable subgroups of patients who are most likely to benefit from a given therapy.

## 1. Background

### 1.1. Epithelial- and High Grade Serous Ovarian Carcinoma

Ovarian cancer is usually diagnosed at an advanced stage due to the late onset of symptoms, which makes its curative care challenging. Almost 314,000 women are diagnosed worldwide with ovarian cancer and more than 200,000 die from the disease every year (https://www.wcrf.org/cancer-trends/ovarian-cancer-statistics/; accessed on 1 October 2022). About 90% of ovarian cancers are of epithelial origin and are thus called epithelial ovarian cancers (EOC). There are several ovarian cancer subtypes, with up to 80% of patients diagnosed with an EOC subtype of ovarian high-grade serous carcinoma (HGSC). The current EOC standard-of-care (SOC) is surgery combined with a platinum and taxane-based chemotherapy. About 80% of patients with advanced cancer respond well to the primary treatment, but unfortunately, almost all of them will relapse and eventually develop a resistant disease [1]. This leads to a short life expectancy, with an overall 5-year survival rate of 42% [2]. The relapsed chemo-resistant HGSC is very aggressive, fast-growing and invasive [3]. Ovarian cancer deaths are expected to increase globally up to 67% by the year 2035, due to an overall increase of the ageing population [4], if no progress in treatment modalities is achieved. In this review, we will concentrate on HGSC and on the recent research concerning its potential treatment with small-molecule kinase inhibitors.

### 1.2. Development of the Current Treatment

The standard first-line treatment of HGSC is cytoreductive surgery combined with platinum and taxane-based chemotherapy. Whether the surgery is completed before or after the chemotherapy depends on the extent of the cancer spread and general health of the patient. The use of platinum compounds as a chemotherapy of ovarian cancer was already introduced about 30 years ago: firstly, cisplatin as a monotreatment [5], and two decades later in combination with taxane [6,7]. While platinum compounds cause DNA crosslinking that modify DNA structure and inhibit its synthesis, taxane compounds prevent microtubule depolymerization, resulting in the inhibition of mitosis and induction of programmed cell death of dividing cells. In the current clinical practice, carboplatin has often replaced cisplatin due to its lower toxicity.

The first targeted treatment of HGSC was the humanized monoclonal antibody bevacizumab that inhibits the binding of the vascular endothelial growth factor-ligand (VEGF) to the VEGF receptor (VEGFR) [8]. Inhibition of VEGF pathway can alternatively be achieved by VEGFR tyrosine kinase inhibitors, such as sorafenib and pazopanib [9]. VEGF pathway inhibition targets tumor vascularization, which is an efficient method to suppress tumor growth and invasion in many cancers, including ovarian cancer, due to its ability to interfere with the high oxygen and nutrition demands of tumors.

Recently, PARP inhibitors, such as olaparib, niraparib and rucaparib, have been introduced as targeted therapy in addition to VEGFR inhibition. PARP1 and PARP2 are needed for the repair of damaged single-stranded DNA. Inhibition of DNA repair with PARP inhibitors induces programmed cell death in cancer cells [8,9]. PARP inhibitors are mostly recommended for relapsed, platinum-sensitive HGSC and are efficient for breast cancer gene type 1 and type 2 (*BRCA*)*1/2*-deficient (germline and somatic) and/or homologous recombination deficient (HRD) tumors, which are expected to cover 20% and 50% of HGSC, respectively.

Both targeted therapy approaches have mainly been used as a maintenance therapy for their ability to slow down tumor growth and metastatic spreading, and they can be administered in combination with chemotherapy and to patients with platinum-sensitive tumors. Trials combining PARP and VEGF inhibition have turned out promising, indicating that their dual targeting could even benefit patients without HRD tumors [10]. Such a synergistic combinatorial effect is likely based on multiple mechanisms, which include the downregulation of homologous recombination regulators *BRCA1/2* and a DNA repair protein RAD51 via VEGFR inhibition-induced hypoxia together with potential *BRCA* downregulation-induced restoration of chemosensitivity [11]. More details about the current treatment recommendations of Food and Drug Administration (FDA), including some more rare and special cases, can be found elsewhere (https://www.cancer.org/content/dam/CRC/PDF/Public/8776.00.pdf; accessed on 1 October 2022).

### 1.3. Challenges in Developing New Treatments

Most targeted cancer treatments are classically designed against growth factors, receptors, cell cycle regulators or other druggable members of signaling pathways that harbor constitutively activated mutations in genes that drive the aberrant growth of cancer cells. Most of these are oncogenes, and their targeting is based on the observation that the cancer cells expressing them exhibit so-called “oncogene addiction”, which manifests in a sensitivity toward a drug or a treatment that targets that particular oncogene or the main signaling pathway it activates [12]. In this respect, HGSC is special since it lacks known driver oncogenes. Instead, a typical driver mutation for HGSC is a loss-of-function mutation of the tumor suppressor *p53* (*TP53*), whose prevalence is close to 100% [13]. Although many experimental approaches have been developed [14], the clinical challenge for the efficient restoration of mutated, inactivated *TP53* still remains.

Immunotherapy has proven very promising for the treatment of many solid tumor cancers. However, it has turned out to be less efficient and more disappointing in the treatment of HGSC. Experimental immunotherapeutic trials have recorded only 4–15% response rates upon targeting the programmed death protein (PD-1) or its ligand (PD-L1) [15], which is poorly expressed in HGSC in general. Of HGSC tumors, generally those that show higher expression of PD-L1 are the *BRCA1/2*-deficient ones, which also typically exhibit higher mutation rates than non-*BRCA1/2*-deficient tumors, and, in this sense, are also more immunogenic. Disappointingly, first trials considering this have shown that *BRCA*-deficient tumors did not demonstrate any better clinical response to PD-1/PD-L1 inhibition either [16]. Despite these obstacles, immunotherapy is still considered a valid possibility for the treatment of HGSC, since ovarian tumors expressing high numbers of T-cells are generally associated with a longer survival, while those showing signs of activated immune evasion mechanisms are associated with a poor survival [15]. Thus, currently, several trials are exploring immunotherapy, namely PD-1/PD-L1 inhibition, in combination with VEGF/VEGFR or PARP inhibition.

### 1.4. Kinase Inhibitors as Cancer Treatments in General

**Figure 1:**
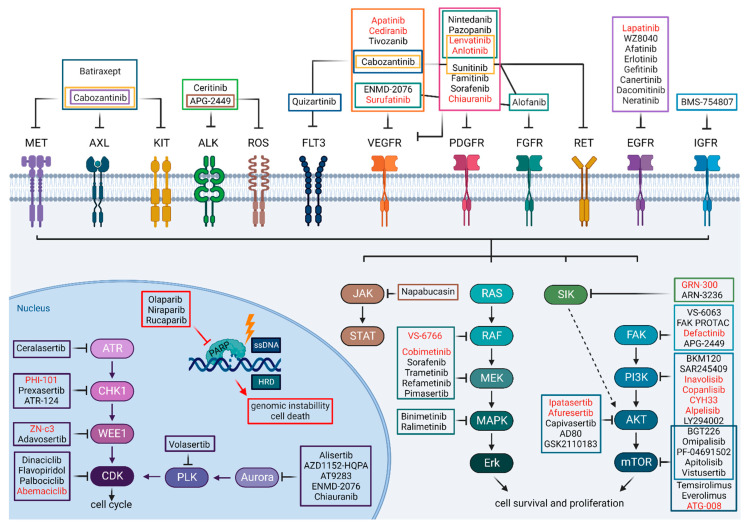
Kinase inhibitors and their targets discussed in this review. Inhibitors highlighted with red color are currently (November 2022) under clinical trials. The colors of the frames around the inhibitors represent the colors used for the kinases they inhibit. Created with BioRender.com.

Deregulated protein kinase signaling is one of the hallmarks of cancer. Moreover, protein kinase families are structurally and functionally similar, making it relatively easy to design and synthesize inhibitors for them. It is, therefore, not surprising that the development of small-molecule kinase inhibitors has revolutionized the cancer treatments [17]. Human kinome comprises 538 kinases and by the year of 2021, 76 kinase inhibitors have received FDA approval as anti-cancer agents (https://www.ppu.mrc.ac.uk/list-clinically-approved-kinase-inhibitors; accessed on 1 October 2022). None of these have been approved for the treatment of HGSC, but several have already been or are currently under evaluation as mono- or combinational therapies for HGSC. In this review, we will focus on small-molecule kinase inhibitors without going into antibody-based inhibition. Figure 1 presents an overview of the kinases and their inhibitors that have recently been or are currently being tested as potential treatments against ovarian cancer.

## 2. Current Progress with Small-Molecule Kinase Inhibitors as Targeted Treatment for HGSC

### 2.1. Many Less and Few More Promising Attempts

The critical cellular processes that are needed for cancer progression, such as increased cell growth and survival, tumor invasion and metastasis formation are regulated by receptor tyrosine kinases (RTKs) via signal transduction from extracellular ligands to intracellular signaling pathways. These ligands include epidermal growth factor (EGF), fibroblast growth factor (FGF), platelet-derived growth factor (PDGF), vascular endothelial growth factor (VEGF), and insulin. The binding of an extracellular ligand to its respective RTK results in receptor aggregation and conformational changes, followed by the phosphorylation of multiple tyrosine residues in its kinase domain and in its C-terminal intracellular domain, leading to its activation. This, in turn, initiates complex intracellular signaling cascades that modulate such diverse processes as proliferation, cell migration, survival, and cell growth. Some of these oncogenic signaling pathways are activated in HGSC [18,19]. Due to high intra and inter heterogeneous nature of ovarian cancer, optimization is needed for the incorporation of kinase inhibitors into clinical practice.

### 2.2. Targeting Receptor Tyrosine Kinases (RTKs)

Since the dysregulation of RTKs is frequent in EOC, and given the pressing need for novel, efficient targeted therapeutics, both single- and multi-kinase inhibitors have attracted significant attention as potential treatments for advanced metastatic ovarian carcinomas.

#### 2.2.1. Aiming at Upregulated ErbB Family Receptors

Epidermal growth factor (ErbB) family of receptor TKs consists of epidermal growth factor receptor (EGFR/ErbB1), ErbB2 (human epidermal growth factor receptor 2, HER2) and ErbB3-4. Immunohistochemical studies indicate that 30–70% of HGSC tumors have increased EGFR expression [20,21], and high EGFR expression has been linked to chemoresistance and poor prognosis [22]. Although small-molecule kinase inhibitors have shown significant clinical benefits in, for example, lung cancers expressing activated EGFR, using these agents as monotherapies had shown a very little effect for HGSC [23]. Consequently, the combination of EGFR inhibitor gefitinib with topoisomerase inhibitor topotecan in HGSC patients did not show sufficient clinical activity either, despite the enrollment of EGFR-positive patients for the trial [24].

Both ErbB2/HER2 overexpression and *ERBB2* gene amplification have been reported in ovarian cancers, and a study on HER2 expression comparing both fluorescence in situ hybridization (FISH) and immunohistochemistry (IHC) staining methods using advanced ovarian tumors from 320 patients indicated that 7% of them were HER2-positive (HER2 3+) [25]. In most studies, elevated HER2-expression has not been associated with prognosis, survival, or treatment response in ovarian cancers, although in some cases, the introduction of HER2 inhibition as antibody-based trastuzumab treatment to the treatment plan has proven efficient [26]. The vast majority of small TKIs targeting either HER2 or both EGFR/HER2 have already been tested in preclinical or phase I trials [27]. Research on the expression of ErbB3 and ErbB4 have not shown significant correlations with disease outcome or clinical variables in EOC either [28]. Despite the reported ErbB4 pathway activation in EOC [29], the use of ErbB4-targeted inhibitors has not reached the level of clinical trials.

#### 2.2.2. Exploiting High Angiogenic Drive

The formation of new blood vessels is essential to sustain continuous tumor growth and metastasis formation. Specifically in EOC, earlier studies have shown high levels of VEGF in ascites, suggesting that peritoneal cavity might be characterized by intense angiogenic activity [30]. Given the fundamental role of angiogenesis in tumor development and the established association of VEGF upregulation with survival, VEGFA-selective antibody bevacizumab was approved for both front-line and maintenance therapy for ovarian cancer [31,32]. Other VEGF-blocking agents, including TKIs, have been investigated in clinical trials, and they seem promising for patients with advanced, relapsed disease. The combinations of selective VEGFR-inhibitors apatinib or cediranib with platinum-based chemotherapy have showed activity and manageable toxicities in several clinical trials [33,34], suggesting that such a treatment combination has potential benefits through therapeutic synergy. Despite the promising results with VEGF TKIs, they have not replaced bevacizumab as a VEGF-targeting approved agent as a first-line treatment for advanced EOC. In the view of abnormal levels of KIT and PDGFR expression found in advanced ovarian cancers, several clinical trials have been conducted with imatinib, which targets both of them [35,36,37,38]. However, imatinib did not show significant clinical activity, neither as a single agent, nor in combination with chemotherapy, nor could the expression levels of PDGFR and KIT predict the treatment response.

#### 2.2.3. Exploring Oncogenic Potential of FGFR

Tyrosine kinase receptors FGFR1-4 (FGFRs) are involved in cell survival, migration, angiogenesis, and carcinogenesis. Both mutations and amplifications in FGFRs are frequent in various cancers, and they are potential ‘driver’ mutations, with FGFR gain-of-function aberrations being strongly related to treatment sensitivity and disease outcome in many cancers [39]. Aberrations in the FGF/FGFR pathway have also been reported in HGSC [39,40,41], with the majority being amplifications or activating mutations, which suggests that FGFR inhibition could be a beneficial therapeutic option for it. The therapeutical targeting of FGFR can be approached with FGFR-selective or multi-targeted TKIs, with the latter ones being already widely involved in clinical trials on ovarian cancer patients.

#### 2.2.4. Probing the Complex Network of IGF Signaling

Insulin-like growth factor (IGF) signaling is needed for the maintenance of healthy ovarian tissue [42]. Hence, the dysregulation of this pathway has been acknowledged in studies involving HGSC [43,44,45]. The insulin-like growth factors IGF1/IGF2, along with the IGF1 receptor IGF1R, play a pivotal role in regulating cell growth, and specifically IGF1R signaling predominates in proliferating cells, being possibly influenced by *p53* status. However, early preclinical studies targeting IGF1R by monoclonal antibodies (mABs) as a monotreatment resulted in a minimal benefit [46], as did the studies using monoclonal antibodies (mABs) in combination with standard chemotherapy or PI3K-AKT/NOTCH/mTOR inhibitors (NCT00718523, terminated prematurely).

The possible reasons for failures of IGF-targeting strategies in the clinical trials of HGSC patients can be rooted to the complexity of IGF signaling. Firstly, to target IGF signaling effectively, one needs to impair the ligand-induced activation of IGF1R while maintaining the control for the insulin-based activation of the insulin receptor (IR) [47]. Secondly, an inefficient targeting strategy may be due to the compensatory signaling by other RTKs, for example, by IR or ERBB family receptors operating outside of the IGF system. Finally, in addition to these direct RTK interactions, the blocking of the IGF1R pathway may be recompensated by the upregulation of downstream signaling converged via canonical PI3K-AKT and extracellular signal-regulated kinase (ERK) cascades [48].

### 2.3. Targeting Intracellular Signaling Cascades

The activation of AKT-PI3K and rapidly accelerated fibrosarcoma and mitogen-activated protein kinase (RAF-MEK) pathways are common in many cancers and can occur by aberrations in upstream signaling molecules, such as RTKs, or via mutations in intrinsic members of the two pathways [49]. The dysregulation of components of these cascades have a prominent effect on cell proliferation, differentiation, and survival. Furthermore, since these pathways are implicated in the resistance and sensitivity to chemotherapy, enormous efforts have been applied to develop inhibitors, specifically targeting the critical components of these pathways, with the aim to increase patient survival and improve response to the standard cancer treatments [49].

#### 2.3.1. PI3K-AKT-mTOR Arm

The PI3K-AKT cascade is one of the best-characterized and most critical signaling pathways with regards to the transduction of anti-apoptotic signals in cell survival, and it is also one of the most frequently aberrated pathways in a range of tumors, including HGSC [50,51,52,53], with *PIK3CA* being increased in copy numbers in 40% and mutated in 12% of HGSC [51,54]. Inhibitors targeting this cascade can be categorized into four groups: PI3K inhibitors, AKT inhibitors, mTOR inhibitors, and dual PI3K and mTOR inhibitors. Despite the clinical trials established for each of these four groups and several PI3K inhibitors being approved by FDA for other cancers, none of the compounds have yet progressed to clinical use for ovarian cancers. Dual PI3K-mTOR inhibitors have not yet advanced beyond phase I in any cancer either, mostly due to the compromised safety or frequent adverse events [55,56,57,58,59].

#### 2.3.2. RAS-RAF-MEK-ERK (MAPK) Arm

The RAS-RAF-MEK-ERK signaling pathway, activated mainly via the ligand stimulation of RTKs, plays a vital role in the diverse cellular processes. Its dysregulation enhances tumorigenesis, impacting not only cell proliferation, but also cell division and survival [60]. The aberrations in the kinases of RAS-RAF-MEK-ERK pathway are frequently observed in various malignancies [61,62,63] including HGSC, where dysregulated activity of this pathway was found in 30% of patients [64]. With regards to HGSC, predominantly MEK and, to a lesser extent, p38 MAPK-selective inhibitors have lately been in the focus of clinical trials phases I-III, but despite great hopes concerning established MEK inhibitors, such as trametinib and selumetinib, their potential usefulness was observed only in the low-grade serous ovarian cancer (LGSC) subtype [65,66], failing to show utility beyond preclinical studies in HGSC [67].

P38 MAPK is another key member of the RAS-RAF-MEK-ERK signaling cascade, which is activated in tumors in response to radiotherapy and chemotherapy. Ralimetinib, a highly potent and selective inhibitor of p38 MAPK, has demonstrated in vivo efficacy in preclinical studies of diverse range of cancer xenografts and cell lines [68,69,70]. This success first inspired a phase I trial in patients with metastatic breast cancer [71], followed by its clinical evaluation conducted in patients with recurrent platinum-sensitive HGSC [72]. However, only a modest improvement in progression-free survival (PFS) was observed [72].

#### 2.3.3. Targeting Cell-Cycle Machinery

Cell-cycle machinery is a tightly regulated series of events enabling cell division. The progression through each stage of the cell-cycle is driven by the proteins called cyclins and their catalytic partners, the cyclin-dependent kinase (CDK) family of serine/threonine kinases. This progression is also strictly monitored at the specific positions known as cell-cycle checkpoints by several cell-cycle checkpoint kinases (CHK) [73]. Hence, it is not surprising that the activities of CDKs and CHKs, being frequent targets for dysregulation in cancer, have led toward the development of the pharmacological inhibitors.

With regards to HGSC, targeting cell-cycle proteins was deemed as a potential strategy, due to the frequent amplification of cyclin E1 (CCNE1) associated with resistance to platinum-based chemotherapy [74]. The aberrant expression of other cyclins, CDKs and CDK inhibitors, has been shown in multiple studies of HGSC [75], suggesting that inhibitors of CDK4/6 might be effective in these tumors. Cell-cycle checkpoint kinases CHK1 and CHK2 are two critical messengers of the genome integrity checkpoints, with CHK1 being especially of interest for the *TP53*-mutated HGSC tumors with a compromised G1 checkpoint [76]. The utility of CHK inhibitors is, however, limited due to the poor safety profile; for instance, cardiotoxicity, including myocardial infarction, has been associated with AZD7762 (CHK1/CHK2 inhibitor; [77]) and MK8776 (CHK1 inhibitor; [78]) in patients with advanced solid tumors.

Mitosis inhibitor protein (Wee1) kinase, phosphorylated and stabilized by CHK1, negatively regulates entry into mitosis at G2/M transition, and, similarly to CHK1, its role in cancer remains controversial. However, Wee1 is upregulated in several cancers, including glioblastoma, melanoma, breast cancer, and ovarian carcinomas, with the latter ones showing higher expression following exposure to chemotherapy [79]. In the preclinical studies, the Wee1 kinase inhibitor adavosertib improved the sensitivity of *TP53*-mutant cells to chemotherapy, which led to its evaluation in clinical trials in patients with *TP53*-mutant HGSC [80,81].

Although the therapeutic potential of cell cycle checkpoint kinases has been in the focus of clinical trials for several years, the development and utility of CHK inhibitors in clinical settings has progressed at a slower rate than for the CDK inhibitors. However, the dysregulated cell-cycle machinery remains an area of intense investigation in ovarian cancer and will hopefully yield new therapeutic modalities in the near future.

### 2.4. Kinase Inhibitors in Recently Concluded Clinical Trials—What Is Promising?

Table 1 presents studies identified by a systematic PubMed search performed on the 5 September 2022. The search gave 368 results, and screening based on title and abstract resulted in 139 relevant papers. To these, the following exclusion criteria were applied: studies published before 2015, studies recruiting several different malignancies, protocol papers, explorative outcome reports, preclinical studies, reviews, breast cancer studies, biomarker profiling studies, and case reports. As most of these clinical studies include patients with ovarian, primary peritoneal, or fallopian tube cancer, the abbreviation OVC is, in this section, used as a collective abbreviation for these histologies. All included studies evaluated clinical responses according to the response evaluation criteria in the solid tumors (RECIST) 1.1 criteria [82].

Forty published clinical studies are included in the final table, with most of them administering kinase inhibitors in combination with other drugs, such as the PARPi olaparib or standard chemotherapy. Twenty-five of the studies reported prolonged progression free survival (PFS) and/or clinical activity of the administered kinase inhibitor, but the conclusions were in general rather modest. One of the more positive studies was performed with apatinib combined with pegylated liposomal doxorubicin (PLD), where both PFS and the overall response rate (ORR) were significantly improved compared to PLD alone. However, the effect was not superior to treatment with PLD combined with bevacizumab [83]. The remaining 15 studies in the table found no effect or even disadvantage of the treatment. The latter was the case for pazopanib maintenance, which decreased OS and increased adverse events (AEs) [99], cabozantinib, which decreased OS, event-free survival (EFS) and showed worse ORR [101], and everolimus, which increased AEs [115].

#### 2.4.1. Multi-Targeted Anti-Angiogenic TKIs

A plethora of phase II-III trials conducted on patients with advanced OVC utilized multi-targeted anti-angiogenic TKIs, such as nintedanib [93,94,95], famitinib [96], pazopanib [97,98,99], sorafenib [100], cabozantinib [101,102], lenvatinib [103], or sunitinib [104], either in combination with other anticancer drugs or as maintenance monotherapy. Even though the majority of these agents showed no additive toxicity, the results of the clinical efficacy of multi-targeted TKIs were vastly discouraging when compared to a standard-of-care platinum-based therapy or maintenance therapy with bevacizumab (Table 1).

The largest study in the table is a double-blind phase III RCT, including 1366 OVC patients treated with a combination of nintedanib and chemotherapy. This results comprise two publications: one reporting the primary outcome, PFS [95], and another reporting the secondary outcome, OS [94]. This study found that while the combination therapy with nintedanib significantly prolonged PFS, the final OS was not affected. Similar results were found in another large phase III RCT with 940 patients with advanced OVC (mostly containing HGSC, but not necessarily excluding other, more rare type of ovarian cancers), where they tested pazopanib as monotherapy [97]. Based on this, it appears that there is still a need for improvement in the treatment strategy with multi-targeted anti-angiogenic TKIs, even though some short-term results might be promising.

#### 2.4.2. Targeting Intracellular Pathways

Most of the completed clinical trials with inhibitors targeting the intracellular signaling pathways have been early phase I trials involving combination studies of PI3K or AKT inhibitors with carboplatin-based or olaparib treatments [108,109,110,120,121] with dose determination, safety, and tolerability explored as primary outcomes. Several studies involving mTOR inhibitors have progressed to phase II [112,113,114,115,122], and most commonly these trials reported the tolerability and safety of the combinational treatments, but the efficacy appeared to be moderate. These efforts suggest that perhaps mTOR inhibitors could show more promising efficiency in ovarian cancer patients whose tumors have alterations in the PI3K-mTOR pathway, and especially when combined with anti-angiogenic agents or chemotherapeutic treatments.

For the inhibition of MAPK signaling, the MEK1-2 inhibitor binimetinib has shown encouraging results in LGSC [105], and in a small phase I study of 34 patients with platinum-resistant ovarian cancer, the clinical benefit of binimetinib was achieved in a subgroup of patients harboring alterations in the MAPK pathway [106]. Ralimetinib in combination with gemcitabine and carboplatin led to the modest improvement of progression-free survival versus chemotherapy alone; however, this study lacked assessment of any molecular profiling, e.g., aberrations in MAPK-signaling pathway or *BRCA* status of the tumors. In light of these outcomes, MAPK inhibition in ovarian cancer warrants further exploration of its role in oncogenesis and resistance to treatment, along with strong rationales to invest in the development of potent inhibitors.

In targeting the cell cycle machinery, adavosertib used in combination with carboplatin and paclitaxel improved first-line chemotherapy in terms of progression-free survival and was relatively well-tolerated [81]. As compared to such promising results in Wee1 targeting, inhibition of ATR, a kinase-regulating CHK1/Wee1 axis and phosphorylating multiple proteins, including RAD51, by a selective agent ceralasertib was investigated in the phase II trial in combination with olaparib, resulting in excellent tolerability but with no objective response in HGSC patients [116]. Polo-like kinase PLK1, which is known to be involved in triggering chromosome segregation and in cytokinesis in general [123], was targeted by the experimental inhibitor volasertib, and the effect was evaluated in a cohort of platinum-resistant ovarian cancer patients, where it demonstrated antitumor activity, along with the manageable side effects [119].

Five of the studies in Table 1 stratified patients according to the relevant genetic alterations of g*BRCA*m [85,91,110], *TP53* [81] and MAPK pathway [106], and four of these found that the patient stratification improves the outcome [81,85,91,106]. This adds to the argumentation that more personalized approaches might be very relevant to consider in future studies regarding the treatment of HGSC with kinase inhibitors.

## 3. Kinase Inhibitors in Ongoing Clinical Trials—What to Expect?

Table 2 includes 29 ongoing clinical trials with kinase-inhibitor treatment of OVC that were posted on ClinicalTrials.gov from 2020 until end of October 2022. Thus, they represent the most recent developments in clinical trials within the field. Studies recruiting patients with various types of advanced solid tumors, and not specifically OVC, were not included in the table.

Despite rather discouraging results achieved with multi-targeted TKIs so far, several of the ongoing trials currently involve lenvatinib (NCT05296512, NCT05422183, NCT05114421, NCT04519151), anlotinib (NCT05145218, NCT04807166, NCT04566952), and surufatinib (NCT05494580).

Of the 29 included studies, 8 take into account either relevant genetic mutations, biomarker expression, or receptor expression in their primary and/or secondary outcomes. One of the ongoing studies uses pathway aberrations, such as *PIK3CA*, as the enrolment criteria (NCT05043922), and a phase III study evaluating the efficacy of the combination of alpelisib and olaparib is aimed at patients diagnosed with HGSC with no germline *BRCA* deficiency (NCT04729387). Germline *BRCA* deficiency is accounted for in a phase II RCT of the VEGFR2 inhibitor apatinib (NCT05479487).

Cobimetinib, a highly selective allosteric MEK1-2 inhibitor, is to be evaluated in the phase II clinical trial of OVC patients with a prior biomarker stratification (NCT04931342), and a study combining VS-6766 (dual RAF-MEK inhibitor), and defactinib has progressed to phase II in both HGSC and LGSC patients with RAS/*BRAF*/NF1 mutations (NCT05512208) or molecularly profiled patients (NCT04625270). Additionally, a study of the salt-inducible kinase 2 and 3 (SIK2- and 3) inhibitor GRN-300 takes genetic variation into account as a secondary outcome (NCT04678102), and a study of the CDK4 and -6 inhibitor abemaciclib combines the treatment with anastrozole for patients with HR+ tumors (NCT04469764).

The ongoing trials mostly administer kinase inhibitors in combination with other drugs and not any more as monotherapy, which was shown to be inefficient in the concluded and published trials. However, in addition to CTX and PARPi, immunotherapy, such as pembrolizumab (NCT04519151, NCT05296512, and NCT05114421), envafolimab (NCT05422183) or durvalumab (NCT04739800), are featured in several new studies. Lastly, only four of the kinase inhibitors (cediranib, apatinib, lenvatinib and afuresertib) included in the ongoing studies are also listed in Table 1, while the remaining 20 ongoing studies in Table 2 use different kinase inhibitors.

These observations indicate that the field is moving toward new strategies in kinase-inhibitor treatment, and with patient stratification and new combination therapy approaches, better results may be achieved in the future.

## 4. Promising Preclinical Studies Using Ovarian Cancer Organoids and Mouse Models—New Arising, Promising Treatments?

### 4.1. Patient-Derived Organoid Cultures as Indicative Model Systems for Preclinical Drug Validation

The discouraging outcome of most clinical drug studies can be partially attributed to a random selection of participants, where specific targeted therapies are directed to patients with diverse genetic backgrounds. The high heterogeneity of HGSC tumors, though, underscores the need for a patient-tailored clinical approach. The patient-derived ex vivo tumor organoid cultures (PDOs) can recapitulate the genetic, histological, and molecular heterogeneity of the primary tumor, thus being an ideal model system for personalized ex vivo testing of drug sensitivity and resistance [74,124]. The studies presented in Table 3 are conducted with the idea of exploring the possibility to utilize HGSC PDOs as a center of clinical decision making before drug administration for either naïve or recurrent patients. However, all of them are rather preliminary due to the low number of samples in conjunction with the lack of patient stratification.

#### 4.1.1. Kinase Inhibition Responses Differ among PDO Cultures

Tumor organoid cultures can mimic primary tumor characteristics and accurately reflect the drug response of the original tumor [125]. In this study, tumor organoids with HRD exhibited similar patterns of drug response, as compared to the organoids that did not harbor HRDgenetic status: organoids carrying *BRCA1* mutation were quite sensitive to PARP inhibitor olaparib and platinum-based drugs. Utilizing only three different PDO cultures, sensitivity toward the VEGFR inhibitor cediranib and the mTOR inhibitor everolimus was demonstrated, while the same PDOs were non-responsive to the VEGFR and EGFR inhibitors, sunitinib and gefitinib, independently of their genetic background. However, in this study, one of the organoid lines was sensitive to another EGFR inhibitor, lapatinib. Pazopanib and trametinib treatment, on the other hand, conferred varying efficiency among the samples [125]. Although this study was highly limited in the number of organoid lines, these observations clearly suggest that drug responses can be varying among PDOs, despite the similar genetic profiles. Interestingly, diverse responses were registered, even after treatment with inhibitors of the same kinase target.

As listed in Table 3, several EGFR inhibitors showed effective anti-tumor response on PDO cultures of which the irreversible pan-EGFR inhibitors canertinib, dacominitib and neratinib have not been part of any clinical trial yet [126]. Similarly, quite high effectiveness was shown with PI3K-mTOR pathway inhibitors, such as omipalisib, PF-04691502 and vistusertib, and with aurora kinase inhibitor alisertib. Targeting the MEK kinase with refametinib exhibited a significant anti-growth effect, while trametinib also turned out to be quite potent [126]. These treatments are assumed to block signaling pathways that promote the renewal of cancer stem cells, which are crucial mediators of tumor progression and chemotherapy escape. However, this study was limited to only three different PDOs.

Personalized treatments with translational potential using PDO cultures are also supported in a study that provides statistically significant correlation of drug doses with clinical response [128]. Characteristically, the effect of platinum and taxane treatment of seven PDO cultures derived from five different patients was comparable with patient’s respective histopathological (chemotherapy response score, CRS), biochemical (CA125) and radiological (RESIST) measurements. Similarly, drug response correlated with the genetic profile in functional assays, as no evident PARP inhibition was reported in any of the 36 *BRCA* gene-inactivated organoids, bearing no HR defects [128]. Moreover, organoids carrying *BRAF*, *KRAS* and *NRAS* alterations were responsive to *BRAF* inhibitor vemurafenib, but not to the pan-HER inhibitor afatinib. Organoids with *TP53* mutations demonstrated inconsistent efficiency patterns toward the Wee1 inhibitor, adavosertib, while organoids with alterations in the *CDKN2A* and *XIAP* genes were responsive to flavopiridol, a CDK inhibitor. Drug screening on one patient´s PDOs that were collected longitudinally (from the chemo-sensitive initial stage or the relapsed chemo-refractory stage) and on PDOs derived from different tumor sites of seven patients further supported the intra-tumor genetic heterogeneity of HGSC and the impact this might cause to SOC treatment [128]. Indicatively, in vitro results with PDOs correlated accurately with the clinical course of the disease. These observations argue for the importance of PDO cultures as a valid material when searching for personalized clinical approaches at specific stages of the disease.

Furthermore, the kinase inhibitors adavosertib, LY294002, sorafenib, capivasertib and trametinib had varying responses in a study where ten different PDO cultures were compared, supporting again the potential usefulness of individualized pre-clinical patient evaluation before medical administration [129]. The inhibitory effect of the Wee1 inhibitor, adavosertib, has also been reported in a study that potentiates the role of this kinase on cell-cycle control and DNA damage response pathways in genetically unstable cancers using two patient-derived ovarian cancer cell lines instead of PDOs [132]. Adavosertib acts via inhibiting cell growth at multiple levels and regardless of the homologous recombination status of the cells. Here lies a potential treatment option for patients that are not susceptible to the current treatments.

#### 4.1.2. Synergistic Effect of Kinase Inhibition and SOC on PARP- or Platinum-Resistant PDOs

In functional assays, using PDOs from ten patients that were insensitive to platinum, indicated that these PDOs were sensitive to such tyrosine kinase inhibitors as the EGFR/HER2 inhibitors, lapatinib and WZ8040, while the use of PI3K and CHEK1 inhibitors, BGT226 and CHIR-124, led to the significant inhibition of tumor progression [127]. In another study using PDOs from a chemoresistant patient, treatment with AXL inhibitor AVB500 resulted in limited tumor survival when used in combination with olaparib, independently of the HR status of the tumor [130]. In addition, the inhibitor had a synergistic DNA-damaging effect with carboplatin and paclitaxel treatment, suggesting that AVB500 treatment could be beneficial in patients both with and without *BRCA* mutations [130]. In respect to DNA damage, the imminent PARP refractory poses an additional challenge, especially for the HR-deficient patients. In preclinical functional assays, PDOs might not respond to PARP inhibitors although their genetic status should indicate otherwise [133]. The complex mechanisms underlying the HR and stalled forks defects impends the further understanding and testing of a wide spectrum of targeted therapeutic drugs. Distinct examples are the ATR and CHEK1 inhibitors, berzosertib and prexasertib, which can be used as agents that induce DNA damage in combination treatments with carboplatin or gemcitabine.

The abovementioned preclinical results may partially explain the unavailing conclusions of the clinical trials, conducted on patient cohorts without prior stratification. Nevertheless, the complex and multiple mechanisms of resistance indicate that patients with a similar mutational background could benefit from different treatment options [128]. As noted in most of the preclinical studies, response to drugs in screening assay varies between different PDOs of different origin. Thus, preclinical testing with PDOs represents a realistic model that could be factored into therapeutic decisions, to test promising treatment options individualized for each patient at a given time point of the disease. An alternative, personalized design of clinical trials based on organoid technology, could be a forthcoming advancement on ovarian cancer management, leading to efficient and meaningful therapies. To use HGSC PDOs for the design of personalized HGSC treatments, the culture conditions should be established to the level that will guarantee both the survival of most of the tumor cultures and retaining their resemblance with the original tumors as close as possible. This may be achievable in the near future due to the recent development of PDO culture techniques for HGSC tumors from 23–38% [134,135] up to 55% in a latest report from 2022 [136].

### 4.2. Lessons to Be Learned from Recent In Vivo Studies Conducted with Xenograft Models

The latest preclinical studies using kinase inhibitors in HGSC tumors in vivo in mouse xenograft studies are depicted in Table 4. Several of these studies already use patient-derived xenograft (PDX) models, which are expected to be the next step for the preclinical testing of therapeutics designed for individual patients. These studies consistently advocate for the engrafted tumors, exhibiting the same genetic, histological and clinical profile as the parent tumor [137,138]. As can be noted from Table 4, almost all published studies reported anti-tumor effects with kinase inhibition in in vivo settings. In most cases, tumor growth was hindered and sometimes metastasis formation was inhibited as well. Kinase inhibitor treatment is mostly used in combination with SOC of platinum and taxane addition or PARP inhibition, a strategy that is expected to prolong disease-free survival. None of the studies specifically reported cancer cell death but reported the inhibition of tumor growth.

#### 4.2.1. Ingenious and Rational Drug Combinations with Kinase Inhibition Should Be Explored

Several studies underlie the potency of the VEGF inhibitor, cediranib, in combination treatments for the inhibition of tumor dissemination and metastasis formation, thus prolonging the overall survival of mice. A synergistic anti-cancer effect with olaparib treatment has been obvious in PDX models [153], regardless of the HR mutational status or PARP-sensitivity of the tumors, and this drug combination has been further supplemented with a ribonucleotide reductase inhibitor triapine, which inhibits DNA synthesis (NCT02466971) [154]. This effort puts forward the idea of a combined mechanistic strategy where, for instance, triapine promotes the *BRCA*ness state, and cediranib enhances DNA damage-induced apoptosis.

Moreover, cediranib and anti-IL6 or anti-PD-1 antibodies have been used together as an effort to overcome possible cediranib-acquired resistance [139]. As different tumors are characterized with distinct gene expression patterns, this study depicts an original and rational example of how combinatory treatments should be designed to not only be more effective, but also aiming to eliminate adverse clinical responses. The selective FGFR2 inhibitor, alofanib, has shown anti-angiogenic and anti-proliferative potential by delaying tumor growth in a dose-dependent manner when administered together in combination with platinum-based chemotherapy [141].

#### 4.2.2. Targeting Focal Adhesion Kinase (FAK) and Anaplastic Lymphoma Kinase (ALK)

An oncogenic role for FAK in HGSC has been suggested, as its kinase activity is linked with tumor metastasis [155] and chemoresistance [156]. As FAK inhibitors as monotherapy have no apparent anti-tumor effect, neither in preclinical models nor in clinical trials, the simultaneous blockage of its FAK-dependent and FAK kinase-independent activities is suggested as an alternative option that could result in more effective potential. Characteristically, the use of the FAK proteolysis targeting chimeric molecule (PROTAC) degrader, which induces degradation of the targeted protein via ubiquitination and proteasome recognition, has proven more reliable in halting tumor growth and metastasis in xenografts than its former analogue, defactinib [143].

Nonetheless, multi-kinase inhibition of the ALK/ROS1/FAK with APG-2449 shows greater DNA damage in taxane-resistant tumors than the FAK-selective defactinib [144]. As acquired resistance is inevitable toward the existing tyrosine kinase inhibitors, novel small molecules need to be developed as drug substitutes. Such an example might be resistance to the ALK inhibitors due to secondary mutations. APG-2449 could be considered an innovative anticancer agent to overcome its primary and acquired resistance and sensitize toward SOC. In the sight of drug repurposing, ALK inhibitor ceritinib sensitizes toward PARP inhibition by causing DNA damage [145], an effect that has not been applied in clinic yet. Even though administration of the drug as a single agent has limited activity on PDX models, combination treatment with olaparib restrained tumor regression significantly and greater in PARP-responsive than in PARP-semi-insensitive tumors [145]. The tolerability of this drug makes its clinical potential even greater.

#### 4.2.3. Multiple Targeting of Cell-Cycle, Cell-Proliferation and Survival Pathways

Pathways mediating cell-signaling, proliferation and survival have been widely studied with respect to HGSC development. Several kinase inhibitors have been proposed as possible treatments against progression of the disease, but with no advent of clinical results. The next experimental and clinical approach might be to target several fragile nodes and functional redundancies to eliminate oncogenic crosstalk. Table 4 presents some studies that suggest using specific kinase activities as biomarkers for studies to overcome chemoresistance refractory. The PI3K-AKT-mTOR pathway is at the center of these approaches. AKT is often overexpressed in aggressive epithelial tumors, indicating that its expression level might even act as a biomarker for disease progression and platinum resistance [157,158]. AKT inhibitor uprosertib provides an additive effect in combination with olaparib when measured as the inhibition of tumor growth. This specific study, however, failed to conclude that kinase inhibition might sensitize toward PARP agents or induce apoptosis [146].

The PI3K-AKT-mTOR pathway represents a tightly controlled signaling pathway that elicits oncogenic signaling under feedback mechanisms, and thus its inhibition is quite challenging [147]. Particularly, efforts to solely block RON, PI3K or AKT activity as monotherapy, presented in Table 4, resulted in the periodical restraint of tumor growth, which was lost after cessation of treatment. On the contrary, combination of the multikinase inhibitor AD80 with RON kinase inhibitor BMS777607 caused a long-term tumor regression and prevented metastasis throughout the 2-week follow-up period. The antitumor effect was even superior to the standard care treatment [147]. These studies suggest that the simultaneous targeting of critical regulators within the same pathway could possibly maximize the anticancer effect via sustained suppression of oncogenic signaling.

Orally available SIK2 inhibitor ARN-3236 can induce apoptosis in cancer cells via parallel inhibition AKT downstream signaling, thus overriding paclitaxel resistance [152]. Moreover, anti-proliferating and anti-apoptotic effects were observed after targeting cyclin-dependent pathways together with AKT inhibition. Correspondingly, dinaciclib-treatment in combination with MK2206 synergized the promotion of tumor regression in xenograft experiments, where established cancer cell lines were grown in immune incompetent mice [151]. Although the tumor development continued during the follow-up period, resistance toward the applied treatment was not detected.

MEK kinase hyperactivation has also been associated with poor prognosis [159] and insensitivity to platinum [160]. Thereafter, the MEK inhibitor trametinib has been considered a promising maintenance therapy agent due to its potency to hinder tumor development. However, the appearance of the stem cell features which promoted cancer progression upon trametinib use [149] suggested that trametinib treatment would be feasible only after its combination with an agent, such as a selective aldehyde dehydrogenase 1A (ALDH1A) inhibitor, that can induce death of the stem cells [161].

In conclusion, even though some studies show encouraging results, such as synergy of prexasertib with olaparib, in inducing DNA damage of both PARP-sensitive and -resistant cancer cells in 14 xenograft models [150], mTOR-induced autophagy and impairment of platinum resistance via TTK inhibition [148], or anti-angiogenic and anti-proliferative potential of the FGFR2 inhibitor alofanib combined with platinum, new potential predictive biomarkers and alternative treatment plans would be imperative for their efficient utilization in ovarian cancer care [141].

## 5. Concluding Remarks and Future Directions

Multiple ex vivo and clinical studies concerning small-molecule kinase inhibitors as potential, novel and efficient treatments for advanced, standard-treatment resistant EOC and HGSC have been carried out during the last years. The concluded and ongoing clinical trials utilizing kinase inhibitors have largely been based on studies conducted with established, commercially available ovarian cancer cell lines and their xenograft models, as well as on the fact that many of these kinase inhibitors have proven highly efficient in other type of cancers harboring activation of the same kinase pathways. The studies that have been testing the selected kinase inhibitors as monotherapy have proven disappointing, which of course reflects well the heterogeneity of the HGSC tumors. Thus, treatments combining specific kinase inhibitors with other kinase inhibitors targeting different signaling pathways or in combination with chemotherapy have shown more encouraging results. However, these studies cannot be expected to lead into a commonly administrable breakthrough treatment due to the heterogeneity of the disease.

One of the major difficulties in establishing an efficient treatment for HGSC is the extensive intratumor heterogeneity that is typical for EOC and HGSC. In addition to this, each patient´s tumors show different genetic aberrations and expression profiles, with practically the only common nominator in HGSC being the loss of the tumor suppressor *TP53*. Both intra- and intertumor HGSC heterogeneities underline the importance of patient stratification and establishment of individualized treatment plans. This could involve the complementary use of PDOs, genomic and RNA sequencing data, while additional clinical trials with combinatorial treatments could also be an advantageous strategy. Nevertheless, the complex and multiple mechanisms of resistance suggest that sometimes patients with similar mutational background could benefit from different treatment options. For these cases, the preclinical testing of combinatorial treatments with PDOs could represent a realistic model to assist therapeutic decisions, to test promising treatment options individualized for each patient at a given time point of the disease. The approaches involving PDOs in clinical decision making would then require the development of culture conditions that enable even better ex vivo survival of patient tumor organoids and faithful perseverance of their original features over the culture period. This could be further supplemented with studies on PDX models. One attractive future possibility could also be the so-called tumor-on-chip culture models, where HGSC organoids or mini-tumors would be cultured together with their matching tumor microenvironment. Those cultures could be set up with microfluidics to mimic tumor vascularization and its utilization in administration of the selected drugs, such as kinase inhibitors, to study their synergistic effects with other treatment options in an ex vivo setup that is as close as possible to the in vivo situation in patients. The development of the ex vivo platform would then significantly speed up the personalized testing of different drug combinations and help in identifying the best option for each patient. Eventually, when enough data on different signaling pathways and their activation status by sequencing and ex vivo testing are collected, the tumor sequencing alone could give enough information for setting up efficient personalized treatment plans.

## Figures and Tables

**Table 1:** Concluded clinical trials with kinase inhibitor treatment of ovarian cancer published since 2015.

	Kinase Inhibitor	Target Kinase	Trial	Patient Group (ITT)	Study Design	Primary Outcomes	Secondary Outcomes	Conclusion	Ref.
Tyrosine kinase inhibitors	Apatinib	VEGFR2	Phase II RCT	Platinum-resistant, progressive, OVC. (n = 152)	1:1 randomization to recieve pegylated liposomal doxyrubicin alone or in combination with apatinib.	PFS	OS, ORR, DCR, and safety	Apatinib plus pegylated liposomal doxorubicin showed promising efficacy and manageable toxic effects.	[83]
			Single arm, phase II trial	Recurrent, platinum-resistant, OVC which failed available standard CTX. (n = 29)	Apatinib administered daily until progression or unacceptable toxicities.	ORR	PFS, OS DCR and toxicity	Apatinib may contribute to achieve clinical benefits with an acceptable safety profile.	[84]
	Cediranib	VEGFRs	Phase III RCT	Platinum-sensitive, recurrent, high-grade serous or endometrioid OVC. (n = 565)	1:1:1 randomization to platinum-based CTX, olaparib or olaparib + cediranib	PFS	Activity within g*BRCA*m or wt subgroups, and PROs	Cediranib + olaparib did not improve PFS and reduced PROs compared to CTX, but had significant clinical activity in patients with g*BRCA*m.	[85]
			Phase II RCT	Platinum-resistant, high-grade OVC. (n = 123)	1:1:1 randomization to (1) weekly PAX, (2) olaparib + cediranib on a continuous schedule, or (3) olaparib + cediranib on intermittent schedule.	PFS and evacuations per day in first four weeks	Compliance, reasons for discontinuation, ORR, OS, and HRQoL.	Cediranib + olaparib showed clinical activity, but was not superior to CTX in terms of PFS.	[86]
			Single-arm, phase II trial	Recurrent OVC with high-grade serous or high-grade endometrioid histology and disease progression on any PARPi. (n = 34)	3 cohorts: platinum-sensitive, platinum-resistant, or progressive disease on PAPRi and subsequent CTX. Olaparib + cediranib on a continuous schedule.	ORR at 8 weeks and PFS at 16 weeks	DCR, safety, and mechanisms of resistance	Cediranib + olaparib was tolerable and showed some activity.	[87]
			Phase I dose escalation trial	Recurrent, advanced breast or gynecologic malignancies. (n = 9, OVC = 7)	3 + 3 design with cediranib + durvalumab + olaparib	RP2D	ORR, PKs, and correlative analyses	The RP2D was tolerable and showed preliminary activity in recurrent ovarian cancer.	[88]
			Double-blind, phase III RCT	Recurrent, platinum-sensitive OVC. (n = 456)	2:3:3 randomization to A: placebo + CTX with placebo maintenance, B: Cediranib + CTX with placebo maintenance, or C: Cediranib + CTX with cediranib maintenance.	PFS comparing arms A and C	OS, toxicity, HRQoL, PFS	Cediranib + CTX with cediranib maintenance improved PFS but had added toxic effects compared to standard treatment.	[89]
			Single-arm, two-stage phase II trial	Recurrent or persistent OVC. (n = 74)	Stratification into platinum-sensitive and platinum-resistant. Both groups recieved oral daily cediranib.	ORR at 16 weeks	PFS, OS and toxicity	Cediranib demonstrated activity. Toxicities were manageable at a reduced dose.	[90]
			Updated analysis of phase II RCT	Recurrent, high-grade serous or high-grade endometrioid OVC or a high-grade histology with a known g*BRCA*m. (n = 90)	1:1 randomization to cediranib + olaparib or olaparib alone with g*BRCA*m and previous anti-angiogenic therapy as stratification factors.	PFS	OS, AEs	Cediranib + olaparib increased PFS versus olaparib alone. OS was increased in patients without g*BRCA*m.	[91]
	Tivozanib	VEGFRs	Single-arm phase II trial	Recurrent, platinum-resistant OVC. (n = 31)	Tivozanib as monotherapy until disease progression or withdrawal.	ORR	PFS, OS, and toxicity assessment.	Tivozanib was effective with moderate toxicity and no treatment-related deaths.	[92]
	Gefitinib	EGFR	Dose escalation phase Ib/II trial	Recurrent or persitent OVC with positive EGFR expression. (n = 19)	Phase Ib: 3 + 3 design with standard dose gefitinib and increasing doses of topotecan. Phase II: 10 patients treated with MTD from phase Ib.	Safety and tolerability	ORR and DOR.	The drug combination was relatively well tolerated, but did not show sufficient clinical activity.	[24]
	Nintedanib	VEGFRs, FGFRs and PDGFRs	Double-blind, phase II RCT	Recurrent OVC. (n = 117)	1:1 randomization to cyclophosphamide + nintefanib or cyclophosphamide + placebo	OS	PFS, ORR, toxicity and HRQoL	Nintedanib + cyclophosphamide did not improve outcomes. More patients than expected remained on treatment for ≥6months.	[93]
			Double-blind, phase III RCT	Newly diagnosed, advanced (FIGO stage IIB–IV) OVC after initial debulking surgery. (n = 1366)	2:1 randomization to CARB + PAX + nintedanib or CARB + PAX + placebo.	PFS and CA125	OS	Nintedanib therapy did not affect final OS results.	[94]
			Double-blind, phase III RCT	Newly diagnosed, advanced (FIGO stage IIB–IV) OVC after initial debulking surgery. (n = 1366)	2:1 randomization to CARB + PAX + nintedanib or CARB + PAX + placebo.	PFS and CA125	OS, time to CA125 progression, AEs, and HRQoL	CARB + PAX + nintedanib significantly increased PFS, but was associated with more gastrointestinal AEs.	[95]
	Famitinib	VEGFR, PDGFR, and KIT	Single-arm phase II trial.	Platinum-resistant, recurrent OVC. (n = 37)	Camrelizumab + famitinib until disease progression or unacceptable toxicities	ORR	DCR, DOR, TTR, PFS, OS, OS at 12 months, and safety	Famitinib + camrelizumab showed antitumor activity with an acceptable safety profile.	[96]
	Pazopanib	VEGFRs, PDGFRs, KIT and FGFRs	Double-blind, phase III RCT	Advanced OVC, after surgical debulking, without progression after first-line platinum-taxane treatment. (n = 940)	1:1 randomization to pazopanib or placebo as maintenance therapy for up to 24 months.	PFS	OS and safety	Pazopanib prolonged PFS, but was not associated with improved median OS.	[97]
			Double-blind phase IIb RCT	Recurrent or persistent OVC. (n = 106)	1:1 randomization to PAX + pazopanib or PAX + placebo	PFS	AEs, ORR and OS.	Pazopanib + PAX was not superior to PAX alone.	[98]
			Combination of two double-blind, phase III RCTs	East asian patients with advanced OVC without progression after first-line platinum-PAX treatment. (n = 354)	1:1 randomization to pazopanib or placebo as maintenance therapy for up to 24 months.	PFS	OS, safety, and AEs	Pazopanib maintenance showed disadvantage in OS and AEs versus placebo.	[99]
	Sorafenib	VEGFR2/3, *BRAF*, KIT, and PDGFRs	Single arm phase II trial	Recurrent or metastatic OVC. (n = 54)	Stratification by prior or no prior treatment with bevacizumab. Treatment with bevacizumab + sorafenib.	ORR	PFS and toxicity	Bevacizumab + sorafenib did not meet the primary endpoint, but did show some activity in the bevacizumab-naïve group.	[100]
	Cabozantinib	MET, VEGFR2, AXL, KIT, FLT3 and RET	Phase II RCT	Persistent or recurrent OVC. (n = 111)	1:1 randomization to daily cabozantinib versus weekly PAX.	PFS at week 16 and week 32	Toxicities, ORR, OS and EFS	No difference in PFS between cabozantinib and weekly PAX. OS, EFS, and ORR were worse in the cabozantinib group.	[101]
			Double-blind, phase II discontinuation RCT	Progressive OVC. (n = 70)	Patients with SD after 12-week open-label lead-in phase were randomized 1:1 to daily carbozantinib or placebo.	ORR at week 12 and PFS	CA125 response and AEs.	Cabozantinib showed clinical activity. Toxicities were acceptable.	[102]
	Lenvatinib	VEGFRs, FGFRs, PDGFRβ, RET, and KIT.	Phase I dose escalation trial.	Recurrent endometrial, OVC. (n = 26)	5 dose cohorts with an accelerated titration design until DLT. Then accural transitioned to 3 + 3 design for further dose levels.	AEs	OR, PFS, and duration of response.	Lenvatinib + PAX showed tolerable side effects and clinical activity.	[103]
	Sunitinib	VEGFRs, PDGFRs, RET, KIT, CD114, and CD135.	Single arm phase II trial	Recurrent or persistent clear cell ovarian cancer. (n = 30)	Sunitinib every day for 4 weeks in 6-week cycles until disease progression or prohibitive toxicity.	PFS at 6 months and clinical response	OS	Sunitinib showed minimal activity as second- and thrid-line treatment.	[104]
Tyrosine and serine/threonine kinase inhibitors	Binimetinib	MEK1/2	Phase III RCT	Recurrent LGSC. (n = 303)	2:1 randomized study of binimetinib versus CTX.	PFS	OS, ORR, DOR, CBR, biomarkers and safety	Binimetinib did not show difference in PFS versus CTX.	[105]
			Dose-escalation, phase Ib trial	Platinum-resistant- or refractory OVC. (n = 34)	3 + 3 design for dose escalation of binimetinib on continuous or intermittent schedule. Additionally 12 patients were enrolled in each group after RP2D determination.	RP2D	Predictive biomarkers of clinical activity (by NGS), CR, PR, ORR and SD.	Binimetinib + PAX was tolerable and RP2D was determined. ORR was modest, but higher in patients with genetic alterations affecting the MAPK pathway.	[106]
	ENMD-2076	VEGFRs, FGFRs, FLT3, KIT, and Aurora A	Single-arm, phase II trial	Platinum-resistant or recurrent OCCC. (n = 40)	ENMD-2076 on contiuous schedule until disease progression or unacceptable toxicity.	ORR and PFS at 6 months	Duration of response	ENMD-2076 did not meet the pre-determined bar for efficacy.	[107]
Serine/threonine kinase inhibitors	Ralimetinib	p38 MAPK	Double-blind, phase Ib/II RCT	Platinum-sensitive, recurrent OVC. (n = 118)	Phase Ib: open-label 3 + 3 dose escalation design. Phase II: 1:1 randomization to ralimetinib + gemcitabene + CARB or placebo + gemcitabine + CARB followed by ralimeinib or placebo maintenance.	Phase 1b: RP2D, phase 2: PFS	OS, ORR, CA125, safety and tolerability.	Addition of ralimetinib to gemcitabene + CTX resulted in a modest improvement in PFS.	[72]
	Capivasertib	AKT	Dose expansion phase Ib trial	Recurrent endometrial, triple negative breast, and OVC. (n = 38, OVC = 16)	Olaparib + capivasertib on an intermittent schedule until progression or toxicity.	MTD and RP2D	ORR, SD, and duration of response.	Olaparib + capivasertib showed no serious AEs, and demonstrated durable activity.	[108]
	Afuresertib		Dose escalation phase Ib trial.	Progressive serous OVC following prior platinum-based treatment. (n = 29 for part I and n = 30 for part II)	Afuresertib + CARB + PAX. Part I was a 3 + 3 dose escalation study and part II was a single-arm evaluation of the clinical activity.	Safety and tolerability (part I) and ORR (part II)	CA125 response and PFS	Afuresertib + CARB + PAX showed clinical activity with the MTD of afuresertib defined as 125 mg/ml.	[109]
	BKM120	PI3K	Dose escalation phase I trial	Recurrent HGSOC or TNBC, or other histology of OVC or BC but with g*BRCA*m. (n = 69; 45 OVC and 24 BC)	3 + 3 design dose escalation study of olaparib + BKM120 with expansion cohorts of 12 patients per cancer type.	MTD and RP2D	AEs	Clinical benefit was observed for both g*BRCA*m and g*BRCA*wt. BKM120 and olaparib can be co-administered with attenuated BKM120 dose.	[110]
	Pimasertib and SAR245409	MEK and PI3K, respectively	Double-blind, phase II RCT	Recurrent LMP or LGSC. (n = 65)	1:1 randomization to pimaserib + SAR245409 or pimasertib + placebo, stratified by tumor histology (LGSOC or LMP/borderline).	ORR	PFS, DCR and AEs.	Pimasertib as single treatment can be alternative to CTX. Pimasertib + SER245409 was not more effective than pimasertib alone.	[111]
	Temsirolimus	mTOR	Two single-arm, single-stage phase II trials	Primary stage III or IV OCCC. (n = 90)	1 cohort form the US and Korea and 1 cohort from Japan recieved CARB + PAX + temsirolimus for 6 cycles or until progression followed by temsirolimus consolidation therapy.	PFS at 12 months	OS, PFS and AEs.	PFS at 12 months, was not increased compared to historical controls. The treatment was well tolerated.	[112]
			Single arm phase II trial	Progressive OVC following platinum-based CTX. (n = 22)	Temsirolimus every seven days until disease progression, inacceptable toxicities, or withdrawal.	PFS	AEs and OS	Temsirolimus treatment was well tolerated, but did not meet the predefined efficacy criteria. Few patients had long lasting SD.	[113]
	Everolimus		Single-arm phase II trial	Recurrent OVC. (n= 50)	Everolimus + bevacizumab until disease progression or unacceptabel toxicities.	PFS at 6 months	Molecular profiling and AEs.	Everolimus + beavcizumab did not show added clinical activity compared to studies of bevacizumab alone.	[114]
			Double-blind phase II RCT	Persistent or recurrent OVC. (n = 150)	1:1 randomization to bevacizumab + everolimus or bevecizumab+ placebo until progression or toxicity.	PFS	Safety and ORR	Bevacizumab + everolimus did not increase PFS compared to bevacizumab alone, and was associated with higer AE rate and discontinuation.	[115]
	Ceralasertib	ATR	Single-arm phase II trial	Recurrent, high-grade serous OVC. (n = 14)	Ceralasertib + olaparib until progression or toxicity.	Toxicity and ORR.	PFS	Olaparib + ceralasertib was well-tolerated, but ORR was unaffected. Some activity was associated with g*BRCA*1m.	[116]
	Berzosertib		Phase II RCT	Recurrent, platinum-resistant HGSC. (n = 70)	1:1 randomization to gemcitabene alone or gemcitabene + berzosertib.	PFS	OS, ORR, CBR, CR, PR, SD, DOR, CA125, and safety	Gemcitabene + berzosertib increased PFS. No added toxic efects were observed.	[117]
	Prexasertib	CHK1	Phase I trial	g*BRCA*m patients with HGSC, who have previously progressed on PARP-inhibitor. (n = 29)	3 + 3 design with a 7-day lead-in of olaparib followed by intermittent prexasertib + attenuated dose of olaparib.	Safety and tolerability	Preliminary anti-tumor activity and PDs.	Prexasertib + olaparib showed preliminary clinical activity in this patient group.	[118]
	Volasertib	PLK1	Phase II RCT.	Recurrent, platinum-resistant- or refractory OVC. (n = 109)	1:1 randomization to volasertib or non-platinum CTX. Two-step design for early saftey analysis.	DCR at 24 weeks	ORR, OS, PFS, HRQoL, safety, PK and biomarker analysis.	Volasertib demonstrated antitumor activity, and AEs were manageable.	[119]
	Adavosertib	WEE1	Double-blind phase II RCT	Platinum-resistant or- refractory, recurrent OVC. (n = 124)	Stratification into HGSOC and non-HGSOC. HGSOC randomized 2:1 to adavosertib + gemcitabine or adavosertib + placebo, and non-HGSOC recieved adavosertib + gemcitabine.	PFS	ORR, OS, safety and tolerability, *TP53* mutations and *p53* expression.	Adavosertib + gemcitabene extended PFS and OS.	[80]
			Double-blind, phase II RCT	Platinum-sensitive *TP53* mutant OVC. (n = 121)	1:1 randomization to adavosertib + CTX or placebo + CTX.	ePFS, safety and tolerability	PFS, ORR, and OS.	Adavosertib + CTX improved ePFS, clinical benefit was observed depending on *TP53* mutation, and AEs were increased.	[81]

The following terms were used for the search: #1(“Carcinoma, Ovarian Epithelial”[Mesh] OR “Ovarian cancer*”[tw] OR “Ovarian carcinoma*”[tw] OR “ovarian neoplasm*”[tw]), #2(“Protein Kinase Inhibitors” [Pharmacological Action] OR “Protein Kinase Inhibitors”[Mesh] OR “Kinase inhibitor*”[tw] OR “kinase inhibition” [tw]) #3(“Clinical Trial” [Publication Type] OR “Clinical trial*”[tw] OR “patient stud*”[tw]). In this table, the OVC abbreviation stands for epithelial (unless otherwise stated) ovarian, primary peritoneal, or fallopian tube cancer, HGSC being the most common form. Studies were open-label unless stated otherwise. Clinical terms: AEs = adverse events, BC = breast cancer, CA125 = cancer antigen 125, CARB = carboplatin, CBR = clinical benefit rate, CR = complete response, CTX = chemotherapy, DCR = disease control rate, DLT = dose-limiting toxicities, DOR = duration of response, EFS = event-free survival, g*BRCA*m = germline *BRCA* mutation, HRQoL = health-related quality of life, ITT = intention to treat, MTD = maximum tolerated dose, OCCC = ovarian clear cell carcinoma, ORR = objective response rate, OS = overall survival, PARPi = poly ADP ribose polymerase inhibitor, PAX = paclitaxel, PFS = progression-free survival, ePFS = PFS by enhanced RECIST1.1, PR = partial response, PRO = patient-reported outcomes, RCT = randomized controlled trial, RP2D = recommended phase II dose, SD = stable disease, TTR = time to response, wt = wild-type.

**Table 2:** Recent, ongoing clinical trials with kinase inhibitor treatment of ovarian cancer first posted on ClinicalTrials.gov in 2020 or later.

	Kinase Inhibitor	Target Kinase	Trial	Patient Group (EE)	Study Design	Primary Outcomes	Secondary Outcomes	Clinical Trials ID	First Posted
Tyrosine kinase inhibitors	Cediranib	VEGFRs	Phase II RCT	Recurrent platinum-resistant OVC with prior bevacizumab. (n = 164)	Comparison of durvalumab + olaparib + cediranib, durvalumab + cediranib, and olaparib + cediranib to CTX.	PFS	ORR, DOR, OS, AEs.	NCT04739800	2021
	Apatinib	VEGFR2	Phase II RCT	Platinum-sensitive, relapsed, high-grade predominantly serous OVC. (n = 132)	Fluzoparib + apatinib versus fluzoparib monotherapy as maintenance treatment.	PFS in PARPi treated patients	PFS, PFS in g*BRCA*m patients, ORR, DCR, DOR, OS, and AEs.	NCT05479487	2022
			Phase II RCT	High-grade serous or endometrioid recurrent OVC. (n = 142)	Safety-lead-in of fluzoparib + apatinib, exploratory cohort of fluzoparib + apatinib in patients with prior PARPi treatment, and fluzoparib monotherapy cohort as active comparator.	Safety lead-in: DLT and RP2D, phase II: ORR	AEs, PFS, DCR, DOR, RR, and CA125	NCT04517357	2020
			Single-arm, exploratory phase II trial	Treatment-naïve stage II-IV OVC. (n = 58)	Apatinib + abraxane and carboplatin or cisplatinum as first-line treatment.	R0 resection rate and PFS	N/A	NCT04590625	2020
	Lapatinib	HER2/neu and EGFR	Dose-escalation phase I trial	Platinum-resistant OVC. (n = 15)	Lapatinib + PAX therapy tested with 4 different concentrations of lapatinib.	PFS and DLT	∆plasma lapatinib, and ABCB1 expression	NCT04608409	2020
	Surufatinib	VEGFR, FGFR, and CSF1R	Single-arm phase Ib/II trial	Platinum-resistant OVC. (n = 38)	Phase Ib: dose de-escalation schedule with 3 + 3 design administering surufatinib + pamiparib. Phase II: RP2D of surufatinib + pamiparib.	Phase Ib: MTD and RP2D, phase II: ORR	PFS, DCR, DOR, OS, PROs, and safety	NCT05494580	2022
	Anlotinib	VEGFRs, FGFRs, PDGFRs, KIT and RET.	Phase III RCT	Platinum-resistant, recurrent, OVC. (n = 405)	TQB2450 + anlotinib versus PAX as weekly treatment	PFS and OS	PFS at 6 months, ORR, DOR, DCR, OS at 12 months, AEs	NCT05145218	2021
			Single-arm, exploratory phase II trial	Newly diagnosed advanced (FIGO stage III-IV) OVC. (n = 56)	Anlotinib + CARB/PAX as first-line treatment.	PFS	ORR, DCR, OS, AEs	NCT04807166	2021
			Single-arm, exploratory phase II trial	Platinum-resistant, recurrent OVC. (n = 68)	Anlotinib + dose-reduced olaparib until disease progression.	PFS, AEs	ORR, DCR, OS, TFST, and QoL	NCT04566952	2020
	Lenvatinib	VEGFRs, FGFRs, PDGFRs, KIT, and RET	Single-arm phase II trial	Recurrent or persistent OCCC. (n = 31)	Lenvatinib + pembrolizumab until progression of disease or unacceptable toxicity.	ORR and 6-month PFS	PFS, AEs, CBR, OS, median PFS, and median OS	NCT05296512	2022
			Single-arm phase II trial	Platinum-resistant, recurrent OVC. (n = 20)	Envafolimab + lenvatinib + VP-16 for 6 cycles, followed by envafolimab maintenance therapy.	ORR	OS, PFS, DCR, and AEs	NCT05422183	2022
			Randomized phase II trial	High-grade serous OVC. (n = 16)	Pembrolizumab or lenvatinib administered first as monotherapy and then as combination therapy. Cohort A: Lenvatinib as monothrapy, cohort B: pembrolizumab as monotherapy.	T-cell dysfunction and proliferation	ORR, T-cell effector function, and T-cell memory establishment	NCT05114421	2021
			Single-arm phase II trial	Platinum-sensitive, recurrent, OVC (except from low grade tumors and mucinous histology). (n = 24)	Pembrolizumab/lenvitanib for up to 35 21-day cycles.	PFS	ORR, time to next-line therapy, OS, HRQoL, AEs, safety and tolerability	NCT04519151	2020
Tyrosine and serine/threonine kinase inhibitors	Ipatasertib, cobimetinib, abemaciclib, inavolisib, palbociclib	AKT, MEK, CDK4- and 6, PI3K, CDK4- and 6, respectively	Phase II platform study	Persistent or recurrent rare OVC. (n = 400)	Stratificatin into 8 arms depending on biomarker expression: (1) Ipatasertib + PAX, (2) cobimetinib, (3) trastuzumab emtansine, (4) atezolizumab + bevacizumab, (5) giredestrant + abemaciclib, (6) inavolisib + palbociclib, (7) inavolisib + palbociclib + letrozole, and (8) inavolisib + olaparib.	ORR	DOR, DCR, PFS, OS, and AEs.	NCT04931342	2021
	VS-6766 and Defactinib	*BRAF*/MEK and FAK, respectively	Single-stage exploratory, parallel cohort, phase II trial	Endometrioid, MOC, HGSC and cervical cancer patients with RAS/*BRAF*/NF1 mutations. (n = 55)	VS-6766 + defectanib for 3 weeks in 28-day cycles.	ORR	AEs, PFS, DCR, DOR, and OR	NCT05512208	2022
			Phase II RCT	Molecularly profiled recurrent LGSC. (n = 100)	Randomization to either VS-6766 monotherapy or VS-6766 + defactinib combination therapy.	ORR	DOR, DCR, PFS and OS	NCT04625270	2020
Serine/threonine kinase inhibitors	Copanlisib	PI3K	Phase II RCT	Patients with recurrent, platinum resistant OVC with progression on PARPi therapy. (n = 96)	Randomization to (1) Experimental arm: copanlisib + olaparib, or (2) Active comparator arm: PAX or liposomal doxorubicin or topotecan hydrochloride.	PFS	ORR, OS, and AEs	NCT05295589	2022
	CYH33		Single-arm phase II study	Recurrent/persistent OVC with clear cell histology. (n = 86)	CYH33 monotherapy	ORR in patients with PI3KCA hotspot mutations	PFS, OS, biomarker alterations impacting PI3K pathway	NCT05043922	2021
	Alpelisib		Open-label phase III RCT	Platinum-resistant/refractory HGSC with no g*BRCA*m detected. (n = 358)	Randomization to (1) Experimental arm: alpelisib + olaparib, or (2)Active comparator arm: either PAX or liposomal doxorubicin.	PFS	OS, tolerability, PS, ORR, CBR, TTR, DOR, PKs, HRQoL	NCT04729387	2021
	Ipatasertib	AKT	Single-arm phase I/Ib trial	High grade serous OVC, and endometrioid adenocarcinoma. (n = 24)	CARB + PAX for up to 3 cycles + ipatasertib until 24 hours before surgery.	DLT in dose escalation and dose expansion phase	Tumor response	NCT05276973	2022
	Afuresertib		Phase II RCT	High grade serous, endometroid, or clear cell OVC. (n = 141)	Randomization to (1) Experimental arm: afuresertib + PAX, or (2) Active comparator arm: PAX.	PFS	OS, ORR, DOR, DCR, BOR, CA125, PKs, and AEs	NCT04374630	2020
	ATG-008	mTOR	Two-arm phase II trial	High grade relapsed or metastatic serous OVC, endometrial cancer, and cervical cancer. (n = 96)	Assigment to either ATG-008 + CTX or ATG010 + CTX.	ORR	TTR, DOR, DCR, OS, PFS, AEs, and safety and tolerability	NCT04998760	2021
	GRN-300	SIK2- and 3	Single-arm phase I/Ib trial	Recurrent OVC. (n = 64)	Phase Ia: GRN-300 as monotherapy, phase Ib: GRN-300 + PAX	RP2D and AEs	PKs, CBR, PFS, PDs and biomarkers	NCT04711161	2021
	PHI-101	CHK2	Phase I dose-finding trial	Platinum-resistance/refractory OVC. (n = 36)	Accelerated 3 + 3 design of PHI-101 to determine MTD	DLT and MTD	Dose interruption, reduction or termination, PKs, ORR, DCR, DOR, PFS, OS, TTP, genetic variation, AEs.	NCT04678102	2020
	Abemaciclib	CDK4- and 6	Single-arm phase II trial	Recurrent OVC, or recurrent endometrial cancer. (n = 32)	All patients receive abemaciclib. Patients with HR+ tumors also receive anastrozole or letrozole per standard of care.	PFS at 16 weeks	ORR, PFS (up to 1 year), AEs, and CBR	NCT04469764	2020
	ZN-c3	WEE1	Single-arm phase I trial	Advanced ovarian cancer or triple-negative breast cancer. (n = 14)	ZN-c3 monotherapy for up to 12 cycles.	Decrease in pCDK1 and/or Ki67, or pHH3 or PCHK1 in tumor cells, and AEs.	CBR, CBR in ovarian cancer, PFS, OS and time to disease progression	NCT05368506	2022
			Single-arm phase I/II trial	Recurrent, high grade OVC with histologic subtypes of serous, clear cell or endometrial. (n = 138)	ZN-c3 + niraparib combination therapy.	Phase I: DLT, phase II: PFS and ORR	DOR, CBR, ORR, OS, AEs, PROs, and PKs	NCT05198804	2022
			Phase Ib trial	Platinum-resistant OVC. (n = 140)	4 cohorts receiving either ZN-c3 + PLD, ZN-c3 + CARB, ZN-c3 + PAX pr ZN-c3 + gemcitabene.	Safety and tolerability and MTD	ORR, DOR, PFS, CA125, and PKs	NCT04516447	2020
	Chiauranib	Aurora B, VEGFRs, KIT, PDGFRs	Double-blind phase III RCT	Platinum-refractory, resistant, OVC. (n = 376)	Chiauranib + PAX or placebo + PAX for 6 cycles followed by single agent therapy of chiauranib or placebo.	PFS and OS	ORR, DOR, DCR, HRQoL, and toxicity	NCT04921527	2021

The studies presented were found with the following search on ClinicalTrials.gov on the 3 October 2022: ovarian cancer + kinase inhibitor with the filters: not yet recruiting, recruiting, enrolling by invitation, and interventional. This was followed up with additional searches on ovarian cancer + each of the target kinases identified in Table 1. Here the OVC abbreviation stands for epithelial (unless otherwise stated) ovarian, primary peritoneal, or fallopian tube cancer. Additional clinical terms not explained in Table 1: BOR = best overall response, HR+ = hormone receptor positive, TFST = time to first subsequent therapy or death, TTP = time to progression.

**Table 3:** Recent kinase inhibitor studies (published after 2017) utilizing ovarian cancer patient organoids and primary cultures isolated mostly from HGSC tumors.

Kinase Inhibitor			Target Kinase	Combination Treatment	Patient-Derived Organoid Samples	Conclusion	Ref.
Tyrosine Kinase Inhibitors	Cediranib		VEGFR	Monotherapy	HGSOC short-term PDOs (n = 3)	Organoids sensitive to the drug.	[125,126]
	Pazopanib		VEGFR	Monotherapy	HGSOC short-term PDOs (n = 3)	Organoids display different sensitivity towards the drug.	
	Sunitinib		VEGFR	Monotherapy	HGSOC short-term PDOs (n = 3)	No drug sensitivity.	
	Gefitinib		EGFR	Monotherapy Monotherapy	HGSOC short-term PDOs (n = 3) PDOs from ascites or tumor tissue (n = 3).	Organoids display different sensitivity towards the drug. Effective response against cell growth.	
	Lapatinib		EGFR	Monotherapy Monotherapy	HGSOC short-term PDOs (n = 3) Platinium resistant HGSOC PDO (n = 1)	Organoids display different sensitivity towards the drug. Moderate response.	[125,127]
	WZ8040		EGFR	Monotherapy	Platinium resistant HGSOC PDO (n = 1)	Moderate response.	[127]
	Afatinib		EGFR	Monotherapy Monotherapy	PDOs (n = 36) PDOs from ascites or tumor tissue (n = 3).	Low responsivness with intrapatient heterogeneity. Effective response against cell growth.	[126,128]
	Erlotinib		EGFR	Monotherapy	PDOs from ascites or tumor tissue (n = 3).	Effective response against cell growth.	
	Canertinib		EGFR	Monotherapy	PDOs from ascites or tumor tissue (n = 3).	Effective response against cell growth, especially under 3D culture conditions.	
	Dacominitib		EGFR	Monotherapy	PDOs from ascites or tumor tissue (n = 3).	Effective response against cell growth, especially under 3D culture conditions.	
	Neratinib		EGFR	Monotherapy	PDOs from ascites or tumor tissue (n = 3).	Effective response against cell growth, especially under 3D culture conditions.	
	BMS-754807		IGF1R/InsR	Monotherapy	PDOs from ascites or tumor tissue (n = 3).	Highly effective response against cell growth, irrespetive of 2D or 3D cuture conditions.	
	Sorafenib		MEK, ERK, VEGFR, Carboplatin/Paclitaxel PDGFR		HGSOC PDOs from ascites or pleural fluid speciments (n = 10)	Consistent inhibitory effects in low micromolar range. IC50 lower to Cmax acssociated with therapeutic dosage, but variability between subjects.	[129]
	Batiraxcept (AVB- 500)		Chemoresistant POV71-hTERT cell AXL Carboplatin/Paclitaxel, Olaparib culture from ascites (n = 1)			Synergistic effect with chemotherapy.	[130]
	Quizartinib AC220		FLT3 Monotherapy Platinium resistant HGSOC PDO (n = 1)			Moderate response.	[127]
				Monotherapy	HGSOC PDOs from ascites or pleural fluid No consistent sensitivity towards all samples speciments (n = 10) (n = 5).		[129]
Serine/Threonine Kinase Inhibitors		LY294002	PI3K	Cisplatin	MCW-OV-SL-3, established cell line from tumor tissue	Sensitization towards cisplatin.	[126,127,131]
		BGT226	PI3K/mTOR	Monotherapy	Platinium resistant HGSOC PDO (n = 1)	Organoids sensitive to the drug.	
		Omipalisib	PI3K/mTOR	Monotherapy	PDOs from ascites or tumor tissue (n = 3).	Highly effective response against cell growth, irrespetive of 2D or 3D cuture conditions.	
		PF-04691502	PI3K/mTOR	Monotherapy	PDOs from ascites or tumor tissue (n = 3).	Highly effective response against cell growth, irrespetive of 2D or 3D cuture conditions.	
		Apitolisib	PI3K/mTOR	Monotherapy	PDOs from ascites or tumor tissue (n = 3).	Effective response against cell growth.	
		Vistusertib (AZD1152)	PI3K/mTOR	Monotherapy	PDOs from ascites or tumor tissue (n = 3).	Highly effective response against cell growth, irrespetive of 2D or 3D cuture conditions.	
		Everolimus	mTOR	Monotherapy	HGSOC short-term organoid culture (n = 3)	Organoids sensitive to the drug.	[125]
		Capivasertib (AZD5363)	AKT	Monotherapy	HGSOC PDOs from ascites or pleural fluid No consistent sensitivity towards all samples speciments (n = 10) (n = 4).		[125,126,129]
		Trametinib	MEK1, MEK2	Monotherapy Monotherapy Monotherapy	HGSOC PDOs from ascites or pleural fluid No inhibitory effects. speciments (n = 10) Organoids display different sensitivity towards HGSOC short-term organoid culture (n = 3) the drug. PDOs from ascites or tumor tissue (n = 3). Effective response against cell growth.		
		Refametinib	MEK	Monotherapy	Highly effective response against cell growth, PDOs from ascites or tumor tissue (n = 3). irrespetive of 2D or 3D cuture conditions.		
		Adavosertib (AZD1775)	Wee1	Monotherapy Monotherapy Monotherapy	PDOs (n = 36) HGSOC PDOs from ascites or pleural fluid speciments (n = 10) Patient-ascites-derived established cell lines (n = 2)	Low responsivness with intrapatient heterogeneity. Consistent inhibitory effects in low micromolar range. IC50 lower to Cmax acssociated with therapeutic dosage, but variability between subjects. Induced apoptosis and reduced proliferation independently of the HR status of the patient.	[128,129,132]
		Berzosertib (VE822)	ATR	Monotherapy	HGSC short-term organoid culture (n = 10)	Organoids display different sensitivity/resistance towards the drug.	[133]
		Prexasertib	CHEK1	Carboplatin, Gemcitabine	HGSC short-term organoid culture (n = 10)	Sesitive for fork-unstable organoids. Resistant for stable. But, combination with carboplatin or gemcitaine promotes instability.	
		CHIR-124	CHEK1	Monotherapy	Platinium resistant HGSOC PDO (n = 1)	Moderate response.	[127]
		Alisertib	Aurora	Monotherapy	PDOs from ascites or tumor tissue (n = 3).	Effective response against cell growth.	[126]
		AZD1152-HQPA	Aurora	Monotherapy	PDOs from ascites or tumor tissue (n = 3).	Effective response against cell growth.	
		AT9283	Aurora	Monotherapy	PDOs from ascites or tumor tissue (n = 3).	Effective response against cell growth.	
		Volasertib	PLK1	Monotherapy	PDOs from ascites or tumor tissue (n = 3).	Effective response against cell growth.	
		Napabucasin	STAT	Monotherapy	HGSOC PDOs from ascites or pleural fluid speciments (n = 10)	No consistent sensitivity towards all samples	[129]
		Vemurafenib	B-raf	Monotherapy	PDOs (n = 36)	High Responsivness.	[128]
		Flavopiridol	CDK	Monotherapy	PDOs (n = 36)	High Responsivness	

**Table 4:** Recent mouse xenograft studies evaluating kinase inhibitor treatments in in vivo setting.

Kinase Inhibitor		Target Kinase	Combination Patient-Derived Treatment Tissue or Cell Lines		Mice	Conclusion	Ref.
			Olaparib PDX		−	Broad anti-tumor effect irrespective of HR tumor status. Combination treatment reduced tumor metastasis and prolonged overall survival.	[139]
Tyrosine Kinase Inhibitors	Cediranib	VEGF	Triapine, Olaparib OVCAR3, SKOV3 HGSOC mouse orthrotropic cell anti-Il6 antibody, antilines; 30200, 60,577 expressing PD1 antibody Trp53-/-, *BRCA*1-/-,Rb, HGS2		Nude, SCID FVB/NCrl, C57BL/6J	Anti-cancer effect regardlesss of HR status. Combination of anti-angiogenic agents with anti-Il6 or anti-PD1 result in prolonged mouse survival.	[140,141]
	Alofanib	FGFR2	Carboplatin/Paclitaxel	SKOV3	Nude	Delayed tumor growth and proliferation in combination treatment.	[142]
	VS6063, FAK PROTAC	FAK	Monotherapy	OVCAR8	NOD/SCID gamma	FAK PROTAC is more effective than VS6063 in inhibiting tumor growth, migration and invasion.	[143]
	APG-2449	ALK/ROS/FAK	Paclitaxel	PDX, OVCAR3	Nude with NSCLC H3122 CDX, SCID with KARPAS-299 CDX	Adminestered alone or in combination SOC can overcome primary and secondary TKI resistance.	[144]
	Ceritinib	ALK	Olaparib	PDX	SCID	Induces more effective tumor regression in combination treatment with Olaparib.	[145]
	Batiraxcept (AVB500)	AXL	Carboplatin/Paclitaxel	PDX, OVCAR5, OVCAR8	NOD/SCID gamma	Improves response to carboplatin, increased DNA damage	[130]
Serine/Threonine Kinase Inhibitors	Uprosertib (LAE003)	AKT	Olaparib	PDX from platinium-resistant EOC patients with former PARPi treament (n = 5)	Balb/c nude	Combination treatment delays tumor growth with higher efficiency compared to monotherapy.	[146]
	AD80 BMS777607 BKM120 GSK2110183	AKT, S6K1 Ron PI3K pan-AKT	BMS777607 Monotherapy	PDX, OVCAR4 OVCAR3sfRon	NOD/SCID gamma	Superior inhibition of tumor growth and metastasis development than SOC. Anti-tumor activity is hindered after cessation of treatment. Anti-tumor activity is hindered after cessation of treatment. Anti-tumor activity is hindered after cessation of treatment.	[147]
	BAY1217389 CFI-402257	TTK, mTOR	Cisplatin	CAOV3, OV90	Nude	Inhibits tumor growth and increased cisplatin sensitivity via inhibiting autophagy in vitro and in vivo.	[148]
	Trametinib	MEK1/2	Monotherapy	PEO4	NOD/SCID gamma	Reduces the rate of tumor growth in vivo, but corellates with cancer stem-like features.	[149]
	Prexasertib	CHK1	Olaparib	Patient-derived ascites (n = 14)	NOD/SCID gamma	As monotherapy or in combination kills tumors cells with de novo or acquired PARP resistance via DNA damage.	[150]
	Dinaciclib	CDK	MK-2206	OVCAR3, CAOV3	NOD/SCID gamma	Delayed tumor growth in CCNE-1-amplified HGSOC xenografts. Selectivly synergistic effect with MK2206.	[151]
	ARN-3236	SIK2	Paclitaxel	OVCAR8	NOD/SCID gamma	Enhances paclitaxel sensitivity.	[152]
